# Genome, biology and stability of the Thurquoise phage – A new virus from the *Bastillevirinae* subfamily

**DOI:** 10.3389/fmicb.2023.1120147

**Published:** 2023-03-14

**Authors:** Martyna Węglewska, Jakub Barylski, Filip Wojnarowski, Grzegorz Nowicki, Marcin Łukaszewicz

**Affiliations:** ^1^Department of Molecular Virology, Faculty of Biology, Adam Mickiewicz University, Poznań, Poland; ^2^GenXone INC, Poznań, Poland; ^3^Department of Biotransformation, Faculty of Biotechnology, University of Wrocław, Wrocław, Poland

**Keywords:** bacteriophage, *Bacillus*, genome, replication, freezing, storage stability, morphology

## Abstract

Bacteriophages from the *Bastillevirinae* subfamily (*Herelleviridae* family) have proven to be effective against bacteria from the *Bacillus* genus including organisms from the *B. cereus* group, which cause food poisoning and persistent contamination of industrial installations. However, successful application of these phages in biocontrol depends on understanding of their biology and stability in different environments. In this study, we isolated a novel virus from garden soil in Wrocław (Poland) and named it ‘Thurquoise’. The genome of that phage was sequenced and assembled into a single continuous contig with 226 predicted protein-coding genes and 18 tRNAs. The cryo-electron microscopy revealed that Thurquoise has complex virion structure typical for the *Bastillevirinae* family. Confirmed hosts include selected bacteria from the *Bacillus cereus* group–specifically *B. thuringiensis* (isolation host) and *B. mycoides*, but susceptible strains display different efficiency of plating (EOP). The eclipse and latent periods of Thurquoise in the isolation host last ~ 50 min and ~ 70 min, respectively. The phage remains viable for more than 8 weeks in variants of the SM buffer with magnesium, calcium, caesium, manganese or potassium and can withstand numerous freeze–thaw cycles if protected by the addition of 15% glycerol or, to a lesser extent, 2% gelatine. Thus, with proper buffer formulation, this virus can be safely stored in common freezers and refrigerators for a considerable time. The Thurquoise phage is the exemplar of a new candidate species within the *Caeruleovirus* genus in the *Bastillevirinae* subfamily of the *Herelleviridae* family with a genome, morphology and biology typical for these taxa.

## Introduction

The *Bastillevirinae* is a subfamily which groups large myoviruses infecting bacteria from the *Bacillus* genus. Its parent taxon-the *Herelleviridae* family-includes phages preying on other firmicute hosts grouped in subfamilies *Twortvirinae* (infecting staphylococci), *Brockvirinae* (replicating in enterococci), *Jasinkavirinae* (associated with listerias) and *Spounavirinae* (SPO1-like viruses parasitizing bacilli). So far, no lysogenic phage has been observed within the subfamily (Barylski et al., 2020). Thus, bastilleviruses may be promising agents for the biocontrol of pathogenic, toxic or otherwise harmful bacteria. What makes these viruses even more attractive is the fact that some of them can reach their host even when it is embedded within a thick biofilm structure. Indeed, some *Bastillevirinae* phages produce enzymes which break up the mucosal polymers building the biofilm and allow the phage to reach the bacterial cells (Ozaki et al., 2017). This ability may prove useful in combating undesirable bacteria from the *Bacillus* genus (e.g., *B. cereus*) that form resilient biofilms and cause a persistent contamination of industrial surfaces. As contaminants, these ubiquitous microorganisms also play a key role in spoilage of starch-rich products and food poisoning. Moreover, some of these bacteria (e.g., *B. anthracis*) may cause serious infections in humans and animals (Celandroni et al., 2016; Elshaghabee et al., 2017; Remize, 2017).

In our study, we focused on a novel phage from the *Bastillevirinae* subfamily, named Thurquoise. The phage was isolated from soil using *B. thuringiensis* ATCC10792 as a host. We analyzed the genome of the new phage, determined its replication dynamics, host range and its stability under different storage conditions. We believe that the presented results may prove relevant in the context of the possible application of that phage.

## Materials and methods

### Bacteria and phage strains

To culture the Thurquoise phage, we used *B. thuringiensis* strain ATCC10792 as a propagation host.

This phage was isolated from garden soil in Wrocław, Poland (51° 14′ N, 17° 13′ E) as described previously in Krasowska et al. (2015). Briefly, *B. thuringiensis* strain ATCC10792 was cultured in a nutrient broth (Biocorp, Poland) overnight. The soil was soaked with a liter of the overnight host culture *in situ*. After a week, soil samples (1 g) were collected and resuspended in water, shaken (200 rpm/30 min/room temperature) and centrifuged (4,500 rpm/10 min). The supernatant was filtered through a polyethersulfone (PES) 0.22 μm Millipore filter. Using the double-agar layer technique, the bacteriophage was isolated with a needle from an individual plaque. The Thurquoise strain used in experiments was obtained by three rounds of single plaque purification and further confirmed by DNA sequencing.

Inoculum for the phage propagation, titration and growth analyzes was prepared as follows: a glycerol stock of the host bacterium was diluted with Trypticase Soy Broth (TSB) medium in a 1:1000 ratio and incubated at 30°C with vigorous shaking for ~18 h. The stock used in this procedure was a flash-frozen mixture of an overnight bacterial culture (in TSB) and 50% glycerol (1:1).

### Phage propagation

A pure phage stock was prepared using double-layer agar cultures. Briefly, a phage lysate with a titer of about 1×10^10^ PFU/ml to cause a nearly confluent lysis was mixed with 1 ml of the overnight bacterial culture, molten pre-cooled soft agar (TSB broth with 0.6% agarose, 500 ml portioned into 10 ml batches) and spread on the surface of TSA (Tryptone Soy Agar) plates. After an overnight incubation in 30°C, the upper layer was scraped from the plates and resuspended in 1 l of SM buffer. The mixture was vigorously shaken (700 rpm/1 h/30°C/Corning® LSE™ Shaking Incubator), treated with DNase I (0.5 U/ml; 2 h/37°C), centrifuged (3,076 × g/15 min/4°C/Sigma 3-16PK centrifuge) and the collected supernatant was titered.

### Phage purification

Phage particles present in the lysate obtained in the previous step were precipitated by the addition of 100 g of PEG 8000 and 58.4 g NaCl per liter of crude lysate. After an overnight incubation at 4°C (~18 h), samples were centrifuged (15,000 × g/15 min/4°C/Sigma 3 K30 centrifuge) and the pellet was resuspended in a lambda diluent (20 mM Tris HCl, 10 mM MgSO_4_ and 0.1 M NaCl, pH 7.5). The resulting suspension was purified by chloroform extraction, and mixed with CsCl (0.75 g/ml final suspension). The mixture was centrifuged (155,000 × g/21 h/4°C/Beckman Optima™ L-90 K Ultracentrifuge). Finally, the phage-containing band which formed in the gradient was carefully collected and dialysed in Slide-A-Lyzer G2 10K MWCO Cassettes against SM buffer (0.1 M NaCl, 8 mM MgSO_4_, 25 mM Tris–HCl, pH 7.5) with increased salinity (1 M NaCl, 18 h, 4°C), then with standard SM buffer (0.1 M NaCl, 3 h at room temperature). Finally, the concentrated phage solution was filtered-sterilized using 0.45 μm pore size (Millipore) and stored at 4°C for further experiments.

### Phage titration

For routine phage maintenance, one-step growth analysis and stability tests, we used a rapid spot titration method. Briefly, 300 μl of the inoculum culture (see “Bacteria and phage strains”) was mixed with 3–4 ml of molten soft TSB agar (TSB with 0.6% low melting point agarose maintained at 45°C) and evenly distributed across a TSA plate. Once the agar solidified, 5–10 μl droplets of decimal phage dilutions were spotted on the lawn. After an overnight incubation (~18 h) at 30°C, plaques that formed on the droplet-covered areas on the lawn were counted, and the titer was calculated (to calculate the titer we selected plates with 5–100 plaques). To determine the phage concentration after purification and the replication dynamics experiments, we used the “whole plate” method. The procedure is nearly identical to the “spot” assay. The only difference is that phage dilutions (100 μl) are added directly to the bacterial culture, and the whole mixture is added to soft agar and spread across the plate. Plaques are counted across the whole petri dish to calculate the phage titer.

### Cryo-EM sample preparation and imaging

To determine the morphology of the Thurquoise phage, we used Cryo-EM (Cryogenic Electron Microscopy) imaging. In order to observe phages with both contracted and uncontracted tails we visualized either untreated virions suspended in lambda diluent (concentrated as described in “Phage purification”) or forced tail contraction as previously described Fokine et al. (2004). This treatment included 10-fold dilution of phage particles (titer 1×10^10^ PFU/ml) in contraction buffer (3 M urea buffered with 50 mM TRIS–HCl pH 8.0 with 1 mM MgCl_2_) incubation for 2 h, at 4°C and treated with DNase I (30 μg/ml) for 20 min at 30°C. Next, the samples were diluted threefold with contraction buffer and centrifuged (75,000 × g/1 h/4°C/Beckman Optima™ L-90 K Ultracentrifuge). The pellet was diluted in 100 μl sterile ultra-pure water. Regardless of the treatment, the samples were prepared for the visualization by transferring 4 μl of phage suspension on the plasma treated carbon grids. The grid was gently blotted by filter paper to remove excess fluid, and the remaining sample was vitrified by rapidly immersing it in liquid ethane using Gatan Cryoplunge equipment. Each frozen grid was transferred onto Gatan 626 CryoHolder, cooled to −174°C, inserted into the TEM specimen chamber of the JEOL 1400 transmission electron microscope (Nanobiomedical Center Adam Mickiewicz University in Poznań) and visualized at 120 kV. All images were analyzed using ImageJ 1.53 k software with DM3 reader plugin. The size of the observed particles was determined based on at least 20 measurements gathered across more than 3 independent micrographs (conducted in comparison to the magnification scale in Inkscape 1.1.1 software).

### Host range determination

To determine the host range of Thurquoise, we performed an efficiency of plating (EOP) assay. Briefly, the phage was diluted to a titer of approximately 1×10^3^ PFU/ml. 50 μl of diluted suspension was mixed with 300 μl of the overnight culture of the tested bacterial strain and 3–4 ml of molten soft TSB agar (TSB with 0.6% low melting point agarose maintained at 45°C). The resulting mixture was evenly distributed across the TSA plate and left at 30°C for an overnight incubation. After incubation, lawns were examined to determine the number and the appearance of any plaques or lysis zones that formed. If no plaques were observed on the given host, the experiment was repeated with the phage titer increased to approximately 1×10^6^ PFU/ml. All experiments were performed at least in triplicate. The obtained results are represented as a fraction of the titer obtained for the isolation host (*Bacillus thuringiensis* ATCC10792). Tested hosts included different strains of the *B. thuringiensis*, *B. cereus*, *B. mycoides*, *B. velezensis*, *B. subtilis*, *B. pumilus* and *Priestia megaterium* (see Table 1).

**Table 1:** Efficiency of plating of the Thurquoise phage on different host strains (members of *Bacillus cereus* group, *Bacillus* genus in general and related *Priestia* species).

Genus	Species	Strain	Observed titer	EOP
*Bacillus*	*mycoides*	gold 1	131.5 ± 26.4	1 ± 0.20
*Bacillus*	*thuringiensis*	Berliner 1915 (ATCC 10792; DSM 2046)	40.3 ± 3.2	0.31 ± 0.02
*Bacillus*	*thuringiensis*	subsp. Kurstaki	-	-
*Bacillus*	*thuringiensis*	Serovar. israelensis (BGSC 4Q7; Serotype 14)	-	-
*Bacillus*	*thuringiensis*	Serovar. poloniensis (BGSC 4BR1; Serotype 54)	-	-
*Bacillus*	*thuringiensis*	Serovar. morrisoni (tenebrionis; BGSC 4AA1)	-	-
*Bacillus*	*thuringiensis*	Serovar. oswaldocruzi (BGSC 4AS1; Serotype 38)	-	-
*Bacillus*	*cereus*	NR 606	-	-
*Bacillus*	*cereus*	6A5 (ATTC 14579)	-	-
*Bacillus*	*cereus*	PCM 2003	-	-
*Bacillus*	*velezenzis*	BGSC 10A6; DSM 231	-	-
*Bacillus*	*subtilis*	NATTO (NAFM5)	-	-
*Bacillus*	*subtilis*	subtilis (PCM 1949; ATTC 6633)	-	-
*Bacillus*	*subtilis*	Natto KB1 PCM B/00114	-	-
*Bacillus*	*pumilus*	GL-1	-	-
*Priestia*	*megaterium*	BGSC 7A16, QBM 1551	-	-

### Replication and lysis dynamics: Turbidimetric lysis assay

An overnight culture of *B. thuringiensis* host (see “Bacteria and phage strains”) was diluted in fresh, warm (30°C) TSB medium to reach OD_600_ = ~0.18 and incubated at 30°C with vigorous shaking (~200 rpm/Corning® LSE™ Shaking Incubator). When the resulting suspension reached OD_600_ = ~0.4 (corresponding to a ∼1.0–2.0 × 10^7^ CFU/ml), the culture was infected with 500 μl of phage suspension (final phage concentration in a flask 5.0–6.0 × 10^7^ PFU/ml) and incubated at 30°C with vigorous shaking (~200 rpm/Corning® LSE™ Shaking Incubator) for 120 min. After phage addition OD_600_ of bacteria culture was monitored at regular intervals for 120 min: every 15 min during the first 30 min and every 10 min during the next 90 min until the optical density of culture was below 0.1.

The phage titer used in the experiment was determined based on a series of preliminary assays in which we tested a number of MOI (Multiplicity of infection) values to pick one that ensured saturation of the bacterial cells with viral particles with minimal risk of an infection with multiple virions. For the final experiment, we selected the smallest dilution that resulted in rapid, continuous, undisrupted lysis of the whole culture (corresponding to MOI in the range of 2.5–6). All experiments were performed at least in triplicate.

### Replication and lysis dynamics: One-step growth curve

An overnight culture of the *B. thuringiensis* host (see “Bacteria and phage strains”) was diluted in fresh, warm (30°C) TSB medium to reach OD_600_ near 0.09 and incubated at 30°C with vigorous shaking (~200 rpm/Corning® LSE™ Shaking Incubator). When the resulting suspension reached OD_600_ = ~0.2 (corresponding to a ∼ 1 × 10^6^ CFU/ml), 20 ml of the culture was infected with 200 μl of the concentrated Thurquoise phage suspension (from the “Phage purification” step) to reach the final titer of approximately 1 × 10^4^ PFU/ml and incubated at 30°C with shaking for 10 min to allow phage adsorption. After this time, the infected culture was diluted in a pre-warmed TSB medium 10- or 100-fold and incubated at 30°C with shaking (950 rpm/Eppendorf ThermoMixer®). The experiment was run for 120 min while samples were collected in 10 min intervals. From each dilution we took two samples. The first, was treated with chloroform (to reach the final concentration of 5%), vigorously shaken to release virions trapped inside infected cells and placed on ice. The second was titrated with no additional treatment. For both samples, 100 μl of each dilution was used for the “whole plate” titration method. Results are reported as the log-transformed average of four independent replicates (± standard error of the mean).

### Phage DNA isolation

DNA was isolated from CsCl-purified phage concentrate by phenol:chloroform extraction. Briefly, proteinase K was added to the final concentration of 100 μg per ml of phage suspension. The reaction was incubated for half an hour at 56°C, cooled and vigorously mixed with an equal volume of phenol:chloroform mixture (1:1). After a short spin (16,000 × g/1 min/room temperature), the aqueous phase was carefully collected and the extraction procedure was repeated. Following an additional round of extraction with pure chloroform, the DNA was precipitated with 3 volumes of an ice-cold mixture of 3 M sodium acetate and 96% ethanol (1:25). The precipitate was separated from the mixture by centrifugation (20,000 × g/10 min/4°C/Hettich Universal 32R), washed with 75% ethanol and resuspended in sterile, nuclease-free water.

### Genome sequencing and assembly

The genome of the phage was sequenced in the DNA Analysis Laboratory of Polish Center for Technology Development: Łukasiewicz Research Network (Wrocław, Poland). Libraries were constructed using the Illumina Nextera XT DNA Library Prep Kit according to the manufacturer’s instructions. Libraries were purified by adding AMPure XP beads (Beckman Coulter) to the reaction (in the quantity of 0.6 of the volume of the sample) and retrieving the DNA (according to manufacturer’s instructions). The concentration of the amplified DNA was normalized and the sample was loaded onto a MiSeq apparatus (Illumina) to perform 250-nucleotide-long paired-end sequencing.

After removal of the adapter sequences, reads were quality trimmed and randomly subsampled with Trimmomatic GPL v3 to obtain a read set roughly corresponding to 300 times the expected genome size. Prepared libraries were assembled using SPAdes 3.9.0, SOAPdenovo 2.04 and Edena 3.131028. Settings of all tools were optimized to assemble a single continuous pseudo-circular genome. Results of the different approaches were cross-validated by sequence comparisons and assessed for structural similarity to blast hits representing most similar genomes. We also assessed the uniformity of the read pair distribution along the final sequence after mapping the reads back to sequence genome (we obtained mean coverage of 3002.2×) to reveal any misassembled regions.

### Analysis of the physical ends of the phage genome

To determine physical ends of the phage genome, we first predicted putative termini positions based on the analysis of the organization of similar phages (*Bacillus* virus BM15) and inspection of read mapping to the assembled genome. Then, we confirmed that these positions are actually ends of the DNA molecule by primer walking (Sanger sequencing) through them. The sequencing procedure was conducted on the 3130xl Genetic Analyzer (Hitachi, Ltd.) in Molecular Biology Techniques Laboratory (AMU, Poznań) using the following primers:

Thurquoise_end_101 5’-GCCTATGGGGAAAGCTACCAATC-3′,

Thurquoise_end_121 5’-ATATAGCAAAAGGATCGGCGGC-3′;

Thurquoise_end_-103 5’-TTACTTGACACCTCCAAGCTGC-3′;

Thurquoise_end_-140 5’-TGTCTAAATTAGGATCAAGATTTCTGTCAAG-3’.

The reaction was set up using BigDye™ Terminator v3.1 Cycle Sequencing Kit (Applied Biosystems™) that utilizes AmpliTaq Polymerases there is known to add additional adenines at the end of the DNA molecule (Samuelson et al., 2004).

### Genome annotation

Protein-coding genes were predicted using GeneMarkS v4.32 (Besemer et al., 2001), Glimmer 3.02 (iterative training) (Delcher et al., 2007), PRODIGAL v2.6.3 (Hyatt et al., 2010) and ZCURVE 3.0 (Guo and Zhang, 2006). CDSs identified only by a single de-novo prediction tool were discarded, unless they had significant BLASTp hit in the RefSeq database (min e-value: 1e-5) or contained conserved protein domains. Conflicting start codons were resolved based on majority voting of the prediction algorithms (which included BLAST hits). BLASTp alignments, together with conserved domains detected by InterProScan, were used to assign functions to protein products of the predicted genes. Both gene arrangement and predicted functions were subjected to manual curation that included BLAST searches against multiple databases (NCBI nr protein, RefSeq, UniProtKB, UniProt), domain localisation (InterProScan, CD-Search), ribosome binding site inspection and literature review (Altschul and Madden, 1997; Marchler-Bauer and Bryant, 2004; Jones et al., 2014). Non-coding RNA genes were predicted using InfeRNAl 1.1.2 (Nawrocki and Eddy, 2013) and tRNAscan-SE 2.0 server (Chan and Lowe, 2019).

### Preliminary classification of the phage

To determine the putative taxonomic position of Thurquoise, we clustered the phage family within the default genome set available on the ViPTree server. The server uses the Phage Proteomic Tree approach (Rohwer and Edwards, 2002; Nishimura et al., 2017). Since the results clearly indicate that Thurquoise is a member of the *Herelleviridae* family, we based the phylogenomic analysis on the seminal work describing this family (Barylski et al., 2020) (with the inclusion of additional phages from the *Caeruleovirus* genus). Briefly, we re-used orthologous protein clusters from the mentioned paper and added missing orthologs from new phages based on their blast hits and InterProscan domains. The analyzed protein families included herellevirus DNA helicase, tail sheath protein, two different groups of virion proteins (including the major capsid protein cluster), and six conserved proteins with no known function. Members of each homolog cluster were aligned using Clustal Omega with default parameters. The resulting alignments were concatenated and analyzed with the IQ-TREE 1.6.12 pipeline (Nguyen et al., 2015; Chernomor et al., 2016) that allows the rapid selection of the suitable model of sequence evolution, construction of a maximum-likelihood tree and ultrafast bootstrap (UFBOOT) analysis. Using a partitioned model, we ran 100 replicates and selected the final tree with the best log-likelihood score (See Supplementary File S1).

Whole-genome phylogeny was constructed using a Gegenees version 2.2.1 tool (Ågren et al., 2012). This tool computes asymmetric similarity measures between each genome pair based on results of blast comparison and clusters taxa using this measure to infer the BioNJ tree. For both nucleotide (BLASTN) and translated-nucleotide (TBLASTX) comparison, we used a pre-defined “accurate” setting of the program. Average nucleotide identity of related phages was estimated using Viridic v1.0_r3.6 software and previously mentioned Gegenees tool.

### Phage stability tests

To test the influence of the ions on the phage stability, we prepared 10 different storage buffers: standard SM buffer, SM buffer with no MgSO_4_ and variants of this buffer with MgSO_4_ replaced with either KCl, CaCl_2_, MnCl_2_, CsCl, CoCl_2_, FeCl_3_ ZnCl_2_ or CuSO_4_ (8 mM in each case). Purified bacteriophage stock was diluted in each of these buffers to obtain a suspension of ~1×10^6^ PFU/ml. Each sample was diluted at least 100× to minimize the influence of the original buffer. Resulting suspensions were titered at the start of the experiment and weekly for 2 months of storage at 4°C.

To test the ability of the phage to survive freezing, phage concentrates were diluted in standard SM buffer or its variants supplemented with one of the four cryoprotectants: glycerol (15%), gelatine (2%) sucrose (0.5 M) or trehalose (0.5 M). Then, the samples were stored at 4°C and −80°C. Gradients stored at −80°C were frozen at −196°C. Each preparation was titered at the start of the experiment and after each of the four freeze–thaw cycles.

To test the heat stability of the Thurquoise phage, we diluted the phage concentrates (see “Phage purification” section in methods) in standard SM buffer to obtain the initial titer of ~1×10^6^ PFU/ml. Then, the samples were incubated in 20°C, 30°C, 40°C, 50°C or 60°C for an hour. Every 10 min, samples from each temperature were titered. All stability experiments were conducted at least in triplicate.

Statistical analysis was carried out on log10 transformed titers by two-way mixed-design analysis of variance (ANOVA; within time, between treatments) with Bonferroni-corrected post-hoc unpaired two-tailed t-tests. Difference between treatments was considered significant if *p*-values were lower than 0.05. All analyzes were conducted using the Pingouin 0.5.3 Python open-source statistical package (Vallat, 2018).

## Results and discussion

### Plaque morphology

**Figure 1:**
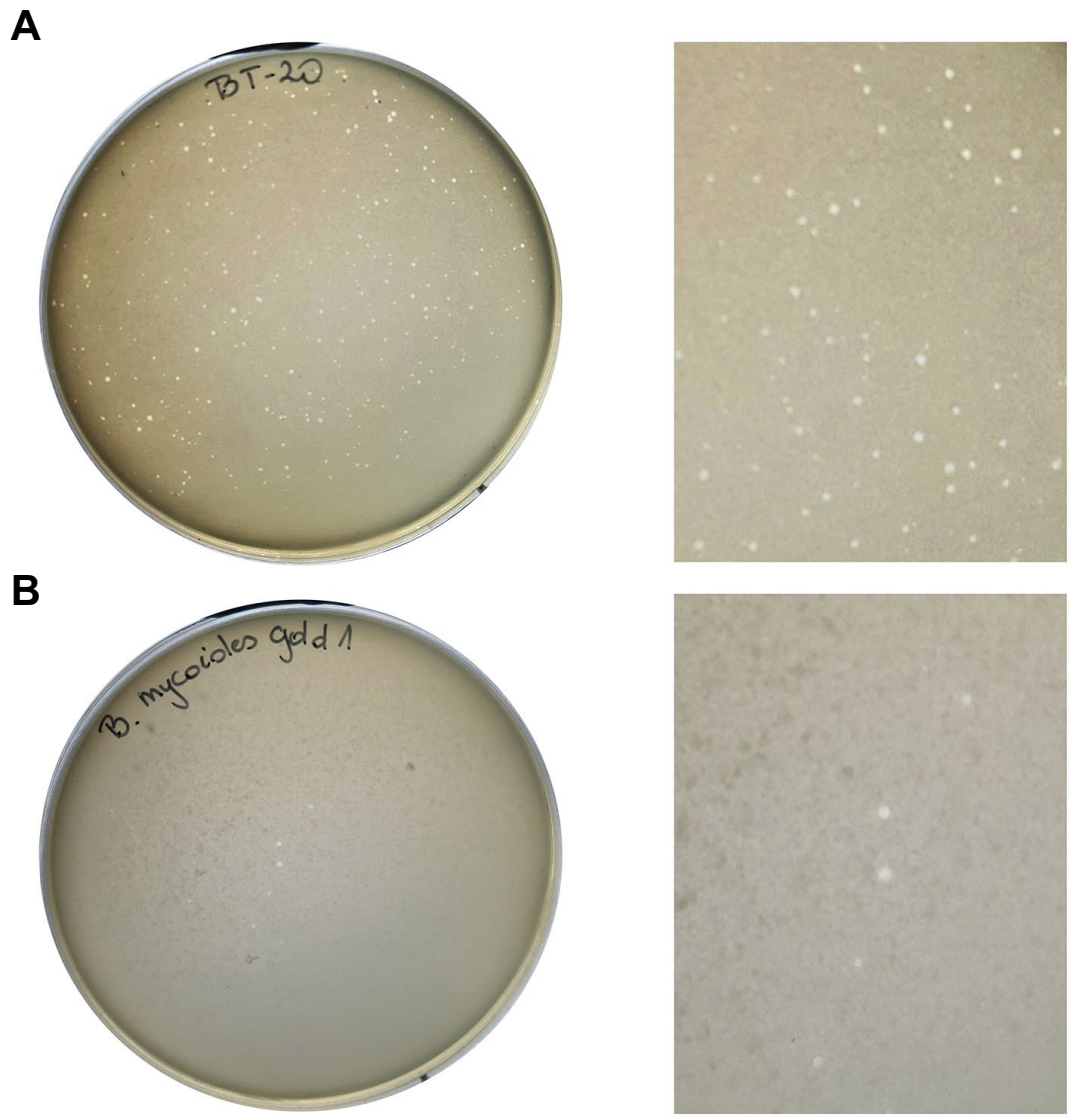
Morphology of plaques formed by Thurquoise phage on *Bacillus thuringiensis* Berliner 1915 **(A)** and *Bacillus mycoides* gold 1 **(B)** lawn growing in 0,6% TSB-based soft agar.

Zones of lysis (plaques) were observed on a bacterial lawn of *B. thuringiensis* Berliner 1915 and *B. mycoides* gold 1 due to the Thurquoise lytic activity (Figure 1). On the double-layer agar culture, on *B. thuringiensis* lawn the formed plaques were hardly noticeable, small in diameter (≤ 0,5 mm), but clear. The plaques formed on *B. mycoides* gold 1 lawn have different morphology. The diameter of zones was larger (0,5–1 mm) and plaques were slightly turbid. In both cases, the best visibility of lytic zones was observed in less concentrated soft agar (0,6%) and the plates were not dried out (closed 5–7 min after preparing).

### Phage morphology

**Figure 2:**
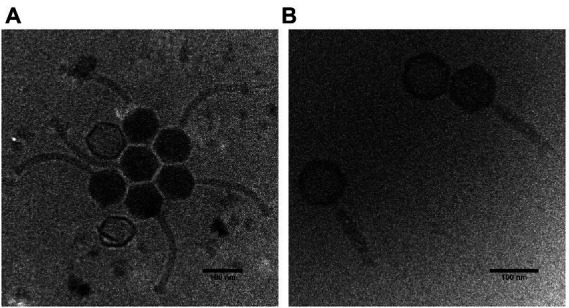
Cryogenic electron micrographs of the Thurquoise virions. Panel **(A)** shows typical morphologies observed under the electron microscope. Panel **(B)** shows phage particles with a contracted tail.

The Thurquoise phage has myovirus morphology with a typical icosahedral head and relatively broad tail (see Figure 2).

Head diameter of this virus (measured between vertices of icosahedron) is 94.3 (± 1.9) nm, while the uncontracted tail is 202.4 (± 27.6) nm long and 14.8 (± 1.4) nm wide. The sheath of the tail can become contracted to 133.8 (± 18.6) nm (simultaneously broadening to 22.2 ± 2.2 nm). The contraction seems to induce the alteration in the tail and baseplate structure that may be similar to changes observed in case of other known herelleviruses (Klumpp et al., 2010; Guerrero-Ferreira et al., 2019; Huang and Xiang, 2020).

Interestingly, many micrographs of the native virus suspended in Lambda diluent show crystal-like clusters of virions with regularly distributed heads. This may indicate that the phage may aggregate in standard phage buffers. On the other hand, this may be an artifact of the sample preparation method as we did not observe similar groupings in the sample with forced tail contraction since (according to our best knowledge) no similar effect was observed in case of any related phage.

### Host range

The efficiency of plating (see Table 1) on the only tested strain of *B. mycoides* seems significantly higher than that observed on the *B. thuringiensis* isolation host (one-tailed *t*-test result <0.05). This might suggest that in natural conditions, the former species is the preferred host for the phage. There is no indication of the phage activity beside these two host species. For a detailed analysis of efficiency of plaiting on different host strains, see Table 1.

### Phage replication cycle

#### Replication and lysis dynamics: turbidimetric lysis assay

**Figure 3:**
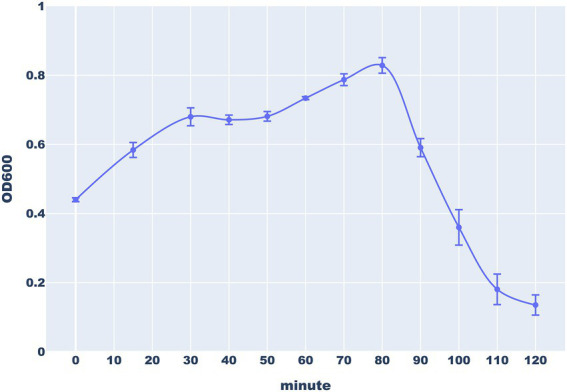
The lysis of *B. thuringiensis* cells by the Thurquoise phage. The culture turbidity was monitored by measuring optical density (OD_600_) of bacterial culture every 15 min during the first 30 min of experiments and every 10 min for the next 90 min. Error bars represent standard deviation from at least three replicates.

To investigate the ability of the Thurquoise phage to lyse *B. thuringiensis*, we monitored the decline of turbidity in the infected culture. The obtained curve showed that Thurquoise completely stops the bacterial growth and starts to lyse the cells ~80 min after infection. The lysis was completed within 120 min of experiment (Figure 3).

### Replication and lysis dynamics: One-step growth curve

To investigate the dynamics of the Thurquoise replication, we conducted the one-step growth experiment. The phage was added to the host culture in the mid-log growth phase at multiplicity of infection (MOI) estimated at 0.01 and allowed to absorb for 10 min. The two samples (treated and untreated with chloroform) were collected in 10 min intervals for 120 min. These two kinds of samples allowed us to differentiate between virion assembly and release.

**Figure 4:**
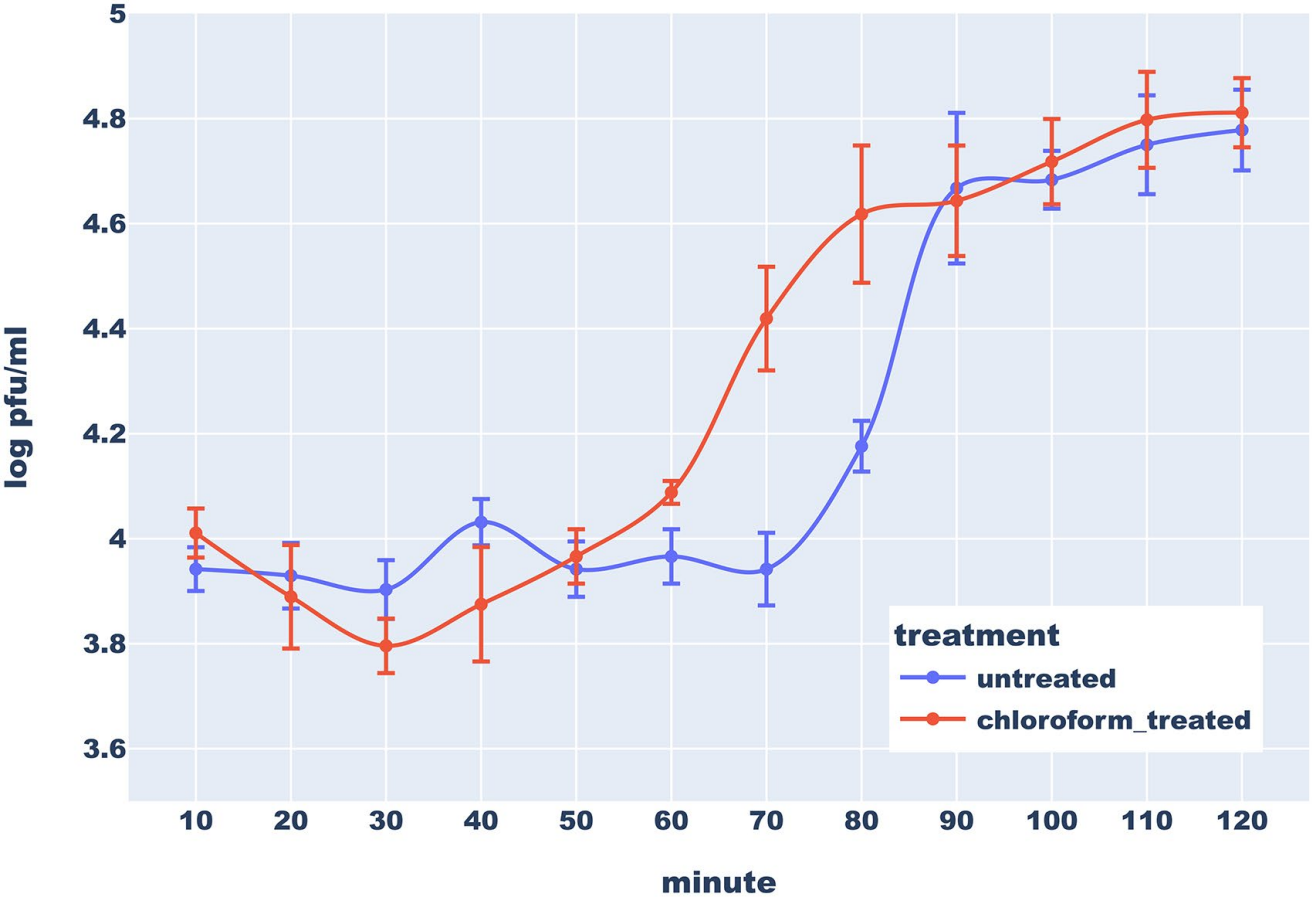
One step-growth curve of bacteriophage Thurquoise in *B. thuringiensis* at 30°C. Results are shown as a change of a titer in untreated (blue line) and chloroform-treated (orange line) samples over 120 min (this duration was determined based on results of similar preliminary experiments – data not shown). Error bars show standard error of the mean (SEM) calculated from log-transformed data.

According to the one-step growth analysis, the eclipse period of Thurquoise lasts ~50 min and the latent period is delayed by 10–15 min. Interestingly, titer in chloroform-treated samples decreased noticeably during the first 30 min. This phenomenon may be explained by the fact that chloroform negatively influences viability of the bacteria before maturation of first progeny virions (Hyman, 2019; Harper et al., 2021). The burst phase starts around 70 min and lasts around 20 min. The average burst size was estimated to be about 7,24 (± 3,19) plaque-forming units (PFU) per infected cell. This result is unexpectedly low for the members of *Caeruleovirus* genus (Shin et al., 2011; Hock et al., 2019) This could be explained in light of the above-mentioned hypothesis that the natural host of the Thurquoise is not the isolated host (strain ATCC10792 *B. thuringiensis*). This conjecture is supported by the fact that the phage forms much larger plaques and has significantly higher efficiency of plating when cultured on *B. mycoides* (Figure 4).

### Analysis of the phage genome

**Figure 5:**
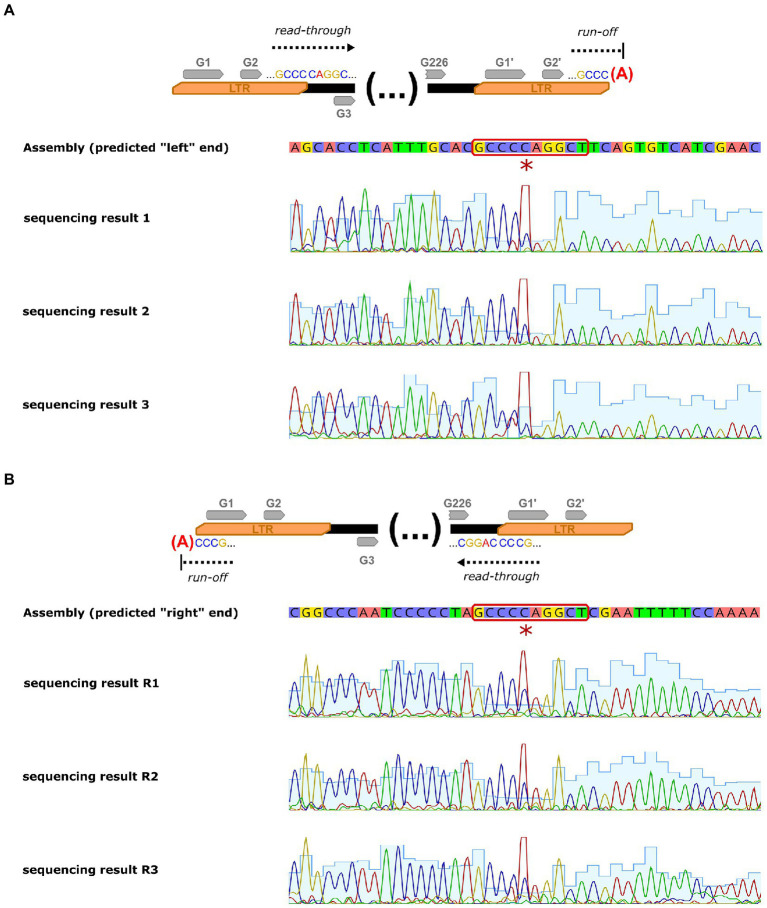
Sanger sequencing of the Thurquoise genome termini. Panel **(A)** shows sequencing determination of the terminus neighboring “right” LTR (arbitrary end of the genome) of the genome while panel **(B)** represents sequencing of terminus neighboring “left” LTR (arbitrary beginning of the sequence). First lane of each panel outlines simplified concept of analysis, the second represents consensus sequence of the genome assembly, while the lanes below are the chromatograms from replicates of the sequencing procedure. The additional non-template adenine added by the sequencing polymerase at the end of the sequence is marked with a red asterisk and the motif repeated at both cutting points is highlighted with a red outline.

The phage genome was assembled into a single gapless contig supported by read mapping. Due to the inherent bias of the Nextera method (non-random read distribution and omission of the physical sequence ends), it is impossible to unambiguously point to the physical ends of the DNA molecule based on the read mapping results. Nevertheless, genomes of similar phages are typically linear and end with long terminal repeats. Based on the analysis of the organization of similar phages and manual inspection of read mapping to the assembled genome, we estimated the size of the repeated regions to ~8 kb; thus, the unique part of the sequence is roughly 157.5 kb long and has a GC content of 39.9%. Our predictions were confirmed by Sanger sequencing of the predicted termini. The sequencing chromatograms contain profound non-template adenines at predicted end positions that have been added at the DNA molecule termini by the polymerase. This clearly indicates that the genome is cleaved in the middle of the inversely repeated “GCCCCAGGCT” sequence (see Figure 5). It may be worth mentioning, that this sequence may theoretically form short hairpin that may be a signal for the terminase to cut (such hairpin may be represented as “(((...))).” in dot-bracket secondary structure notation).

**Figure 6:**
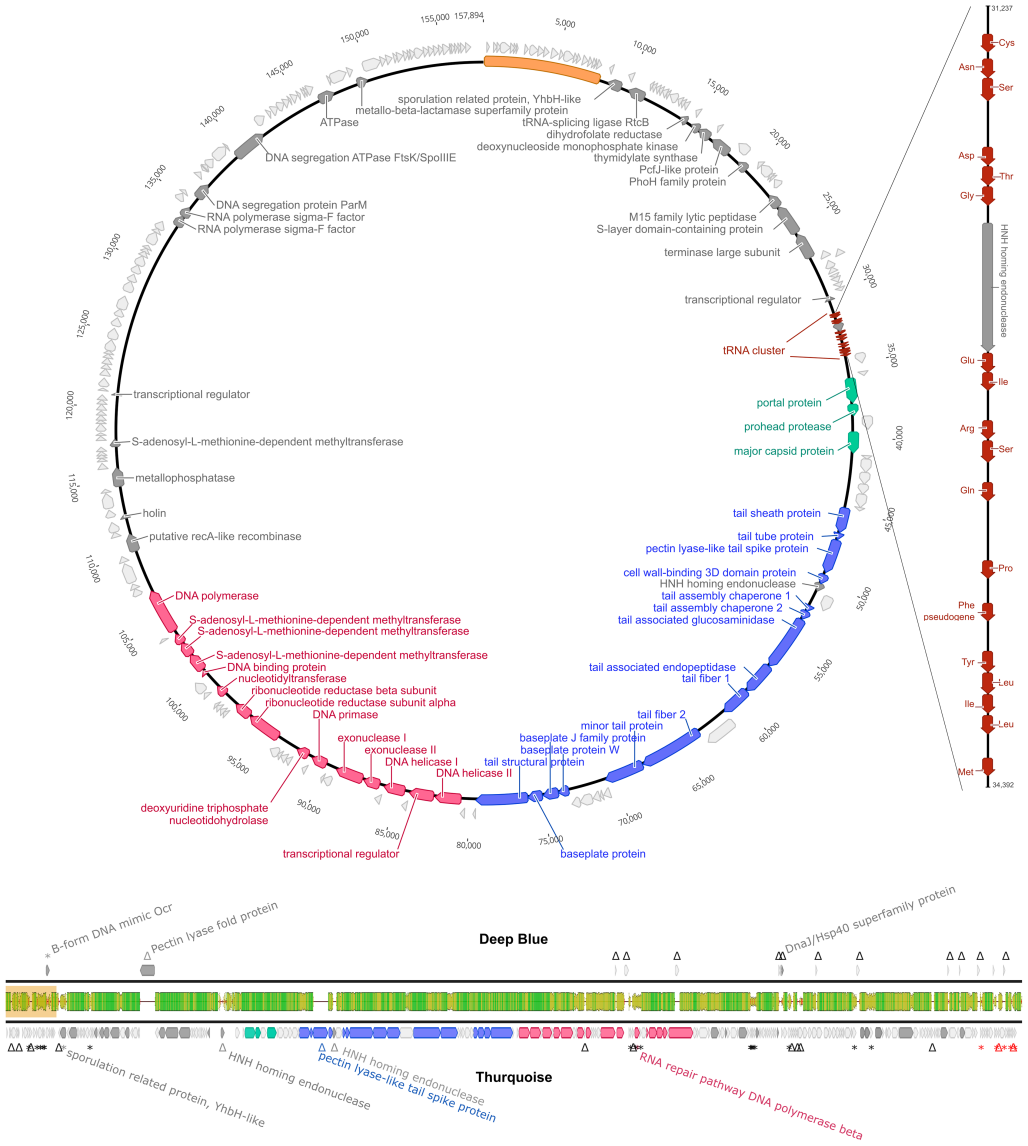
Genome organization of Thurquoise. The top panel shows a simplified circular map of the 157,894 bp-long unique part of the whole 165,897 bp genome (sequence repeated at the ends of the genome is shown as a yellow bar). Coding DNA sequences (CDSs) are colored according to their indicated function: head assembly–green, tail assembly–blue and DNA replication–red. CDSs with no assigned function are light gray. The tRNA genes are marked as smaller cinnabar arrows magnified in the side panel representing the magnified tRNA cluster. The bottom panel is a genome comparison of bacteriophages Thurquoise and its closest known relative–Deep Blue, shown as a pairwise alignment. Arrows indicate the predicted genes (yellow). The middle bar shows the DNA sequence similarity between the two genomes and is colored from green (100% identity) through yellow (~50% identity) to red (less than 10%). Regions with no alignment are shown as a thin black line.

The genome encodes 226 non-redundant proteins and 18 tRNAs. Interestingly, the tRNA cluster was invaded by the mobile HNH nuclease gene that splits one of the genes into two unequal parts. Such elements are usually associated with the group I transposable introns but no indication of typical intronic RNA structures was found during the InfeRNAl analysis (Figure 6).

The phage genome displays a significant sequence similarity to members of the *Caeruleovirus* genus, especially its exemplar–*Bacillus* phage Deep Blue. Yet, regardless of measurement method (Gegenees of Viridic) the average nucleotide identity with other known phage never reaches 95% suggesting that Thurquoise is an isolate of new species (see table S2). The architecture of the Thurquoise genome is typical for the caeruleoviruses. Some functional modules are clearly recognizable, e.g., the long morphogenesis module that can be subdivided into parts encoding the head and tail proteins or ~ 25 kb long stretch of genes associated with DNA replication. The complete annotated genome is available in Genbank under accession number ON975036.

### Preliminary classification of the phage

Our initial intuition based on the blast results was supported by the results from the VipTree classification tool. Bacillus phage Thurquoise seems to be a typical member of the *Bastillevirinae* subfamily and the *Herelleviridae* family.

**Figure 7:**
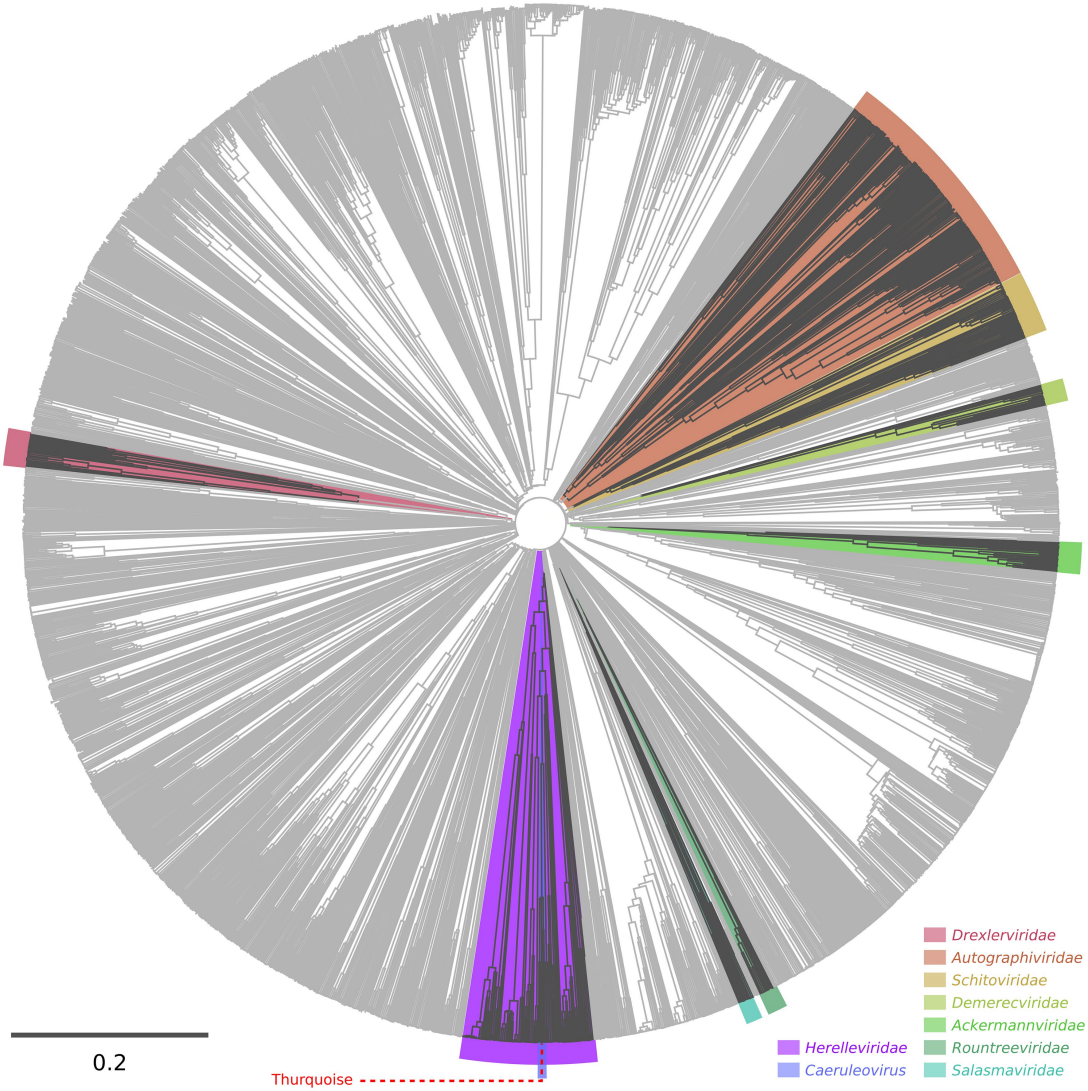
Proteomic tree of phages calculated using the VipTree server. The dendrogram represents proteome-wide similarity relationships among 2,688 viral genomes. The position of the Thurquoise phage is marked with a dashed line. Clades representing parent taxa of the studied virus are highlighted with violet (family) and blue (genus).

**Figure 8:**
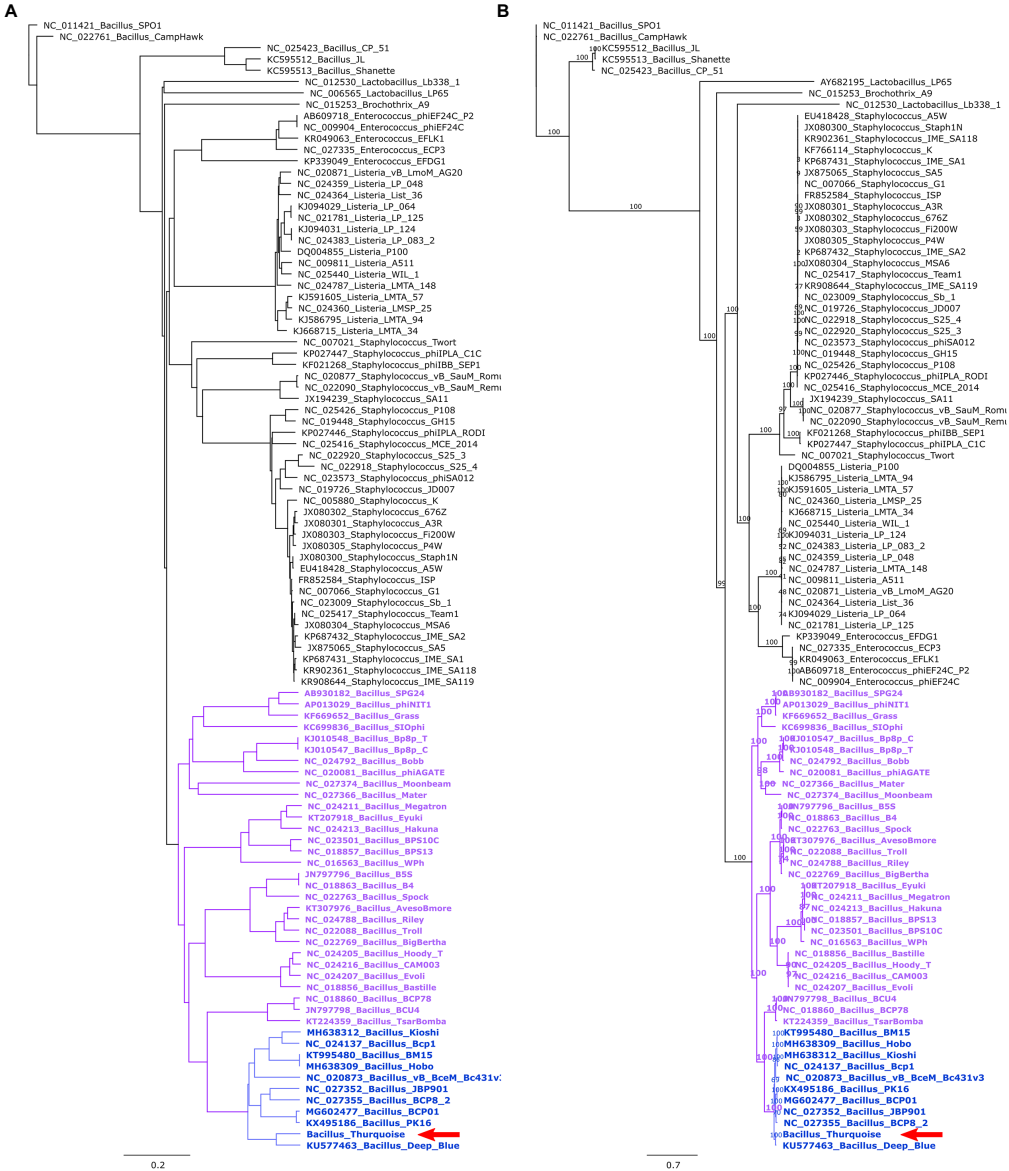
Trees showing the position of the Thurquoise phage in the *Herelleviridae* family. Section **(A)** shows the whole-genome BioNJ tree of the studied viruses. Clustering is based on translated nucleotide similarity and the scale bar represents a 20% difference in the average tBLASTx score. Genomes were compared using Gegenees (tBLASTx method, “accurate” setting set), pairwise similarities were transformed into a distance matrix and clustered using the same tool. Section **(B)** contains the Maximum-likelihood tree based on a concatenated alignment of 10 marker proteins generated using clustal omega and IQ-tree. The scale bar represents the number of substitutions per site, branch support values were calculated from 1,000 ultrafast bootstrap (UFBOOT) replicates. Both trees were rooted at the *Bacillus* phage SPO1–the type species of the family. The position of the Thurquoise phage is marked with a red arrow. Clades representing parent taxa of the studied virus are highlighted with violet (subfamily) and blue (genus).

A detailed phylogenetic analysis shows that the Thurquoise phage may be a candidate member of the *Caeruleovirus* genus. This classification is congruently supported by all applied phylogenetic methods. The results of these methods are shown in Figures 7, 8 (BLASTN-based gegenees tree is available in Supplementary File S1).

### Phage stability

**Figure 9:**
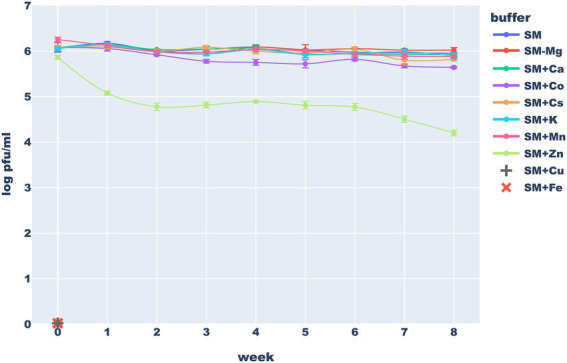
Long-term stability of the Thurquoise phage in different storage buffers. Results are expressed as log_10_ (PFU/mL). Data points represent the mean of three independent experiments and error bars show standard error of the mean (SEM) calculated from log-transformed data.

Typically used storage buffers seem well suited for mid-term storage of the studied phage. After 2 months at 4°C, only minimal titer loss was observed in the SM buffer and variants of this buffer in which magnesium was completely removed or replaced with cesium (commonly used during phage purification), manganese, calcium or potassium (often present in drug formulations). The titer of these solutions remained above or near 50% of the initial value even after 2 months of storage. Interestingly, the stability in the SM with CsCl was comparable to this in the standard version of this buffer–after 60 days more 30% virions remain viable (a difference that was not statistically significant). This suggests that minimal carryover of cesium used in lab-scale phage purification methods is not very harmful for phage particles (it is still unacceptable in any consumer-targeted phage products, though). On the other hand, replacing the Mg^2+^ in the SM buffer with Zn^2+^ or Co^2+^ ions caused a significant drop in phage titer while the addition of Fe^3+^ and Cu^2+^ ions caused a complete inactivation of the phage. We hypothesized that the observed effect e of FeCl_3_ may be at least partially caused by phage precipitation while zinc ions are known for antimicrobial properties thus, they might have influenced the host. The stability of viable phage particles in various buffers is presented in Figure 9.

The inactivating effect of 1 mg/ml of Fe^3+^ ions on various unrelated phages was previously noticed by Raza et al. (2022). Similar, but weaker activity of 5 mM Cu^2+^ ions, was observed against dsDNA phages by Li and Dennehy (2011). Armstrong et al. (2017) suggest that inactivation of viruses by copper ions require hours or even days, thus the effect that we observed may be more profound simply because of longer exposure times. Finally Thorne and Holt (1974), working with another herellevirus – *Bacillus* phage CP-51 showed stabilizing effects of Mg^2+^, Ca^2+^, or Mn^2+^ ions. Our results suggest that comparable concentrations of these ions have little effect on the long-term phage viability.

**Figure 10:**
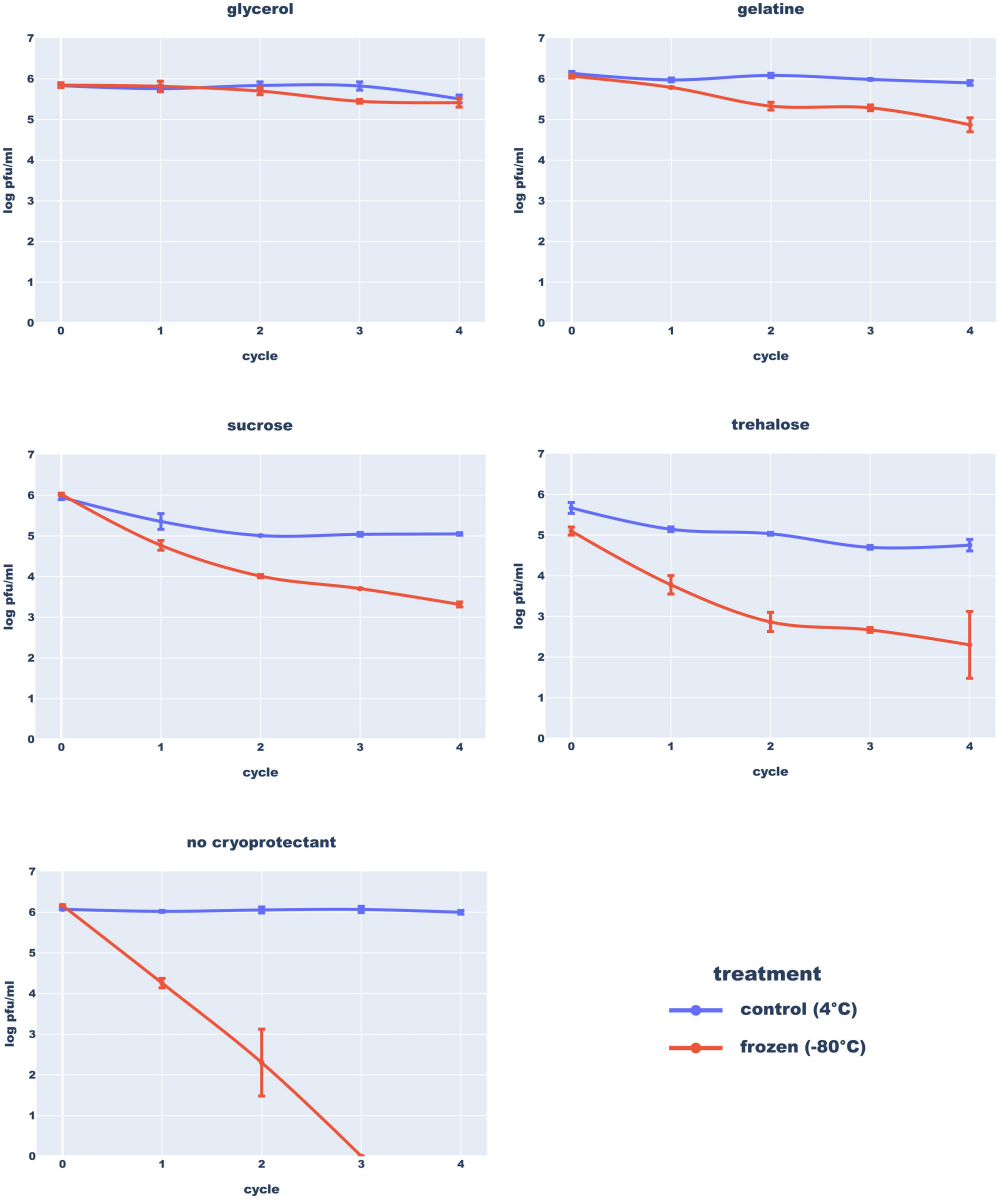
Resistance of the Thurquoise phage to freezing in different buffers. Results are expressed as log_10_ (PFU/mL). Data points represent the mean of three independent experiments and error bars show standard error of the mean (SEM) calculated from log-transformed data.

Results of the freezing test (Figure 10) clearly demonstrated that long-term storage of the Thurquoise phage in temperatures below 0°C requires a proper cryo-protection. In SM, the titer dropped below 1% of the initial value after the first freeze–thaw cycle. This drop was mitigated by the use of any of the studied cryoprotectants. The highest survival rate was observed in samples supplemented with 15% glycerol flash-frozen at −196°C and stored in −80°C. The addition of 2% gelatine provided a slightly lower protection but both results were statistically significant. The effect of sucrose and trehalose was not so profound (and is not supported by outcome of statistical tests). Moreover, addition of these sugars resulted in immediate, significant drop of the titer in both 4°C and −80°C suggesting that they may have a negative impact on phage viability of transmission. All results considered, storage at 4°C may be favorable for a shorter period of time as previously reported for phage MS2 (Olson et al., 2004).

Willms et al. (2017) studied the stability of two other *Bacillus-infecting* phages from *Herelleviridae*–vB_BsuM-Goe2 and vB_BsuM-Goe3. They observed that vB_BsuM-Goe2 and vB_BsuM-Goe3 are relatively stable in 20°C and 4°C but deep-freezing of stocks caused massive titer drop, even in the presence of glycerol. Results of the Thurquoise experiments indicate that his phage can survive freezing, but only in the presence of the propper cryoprotectant.

**Figure 11:**
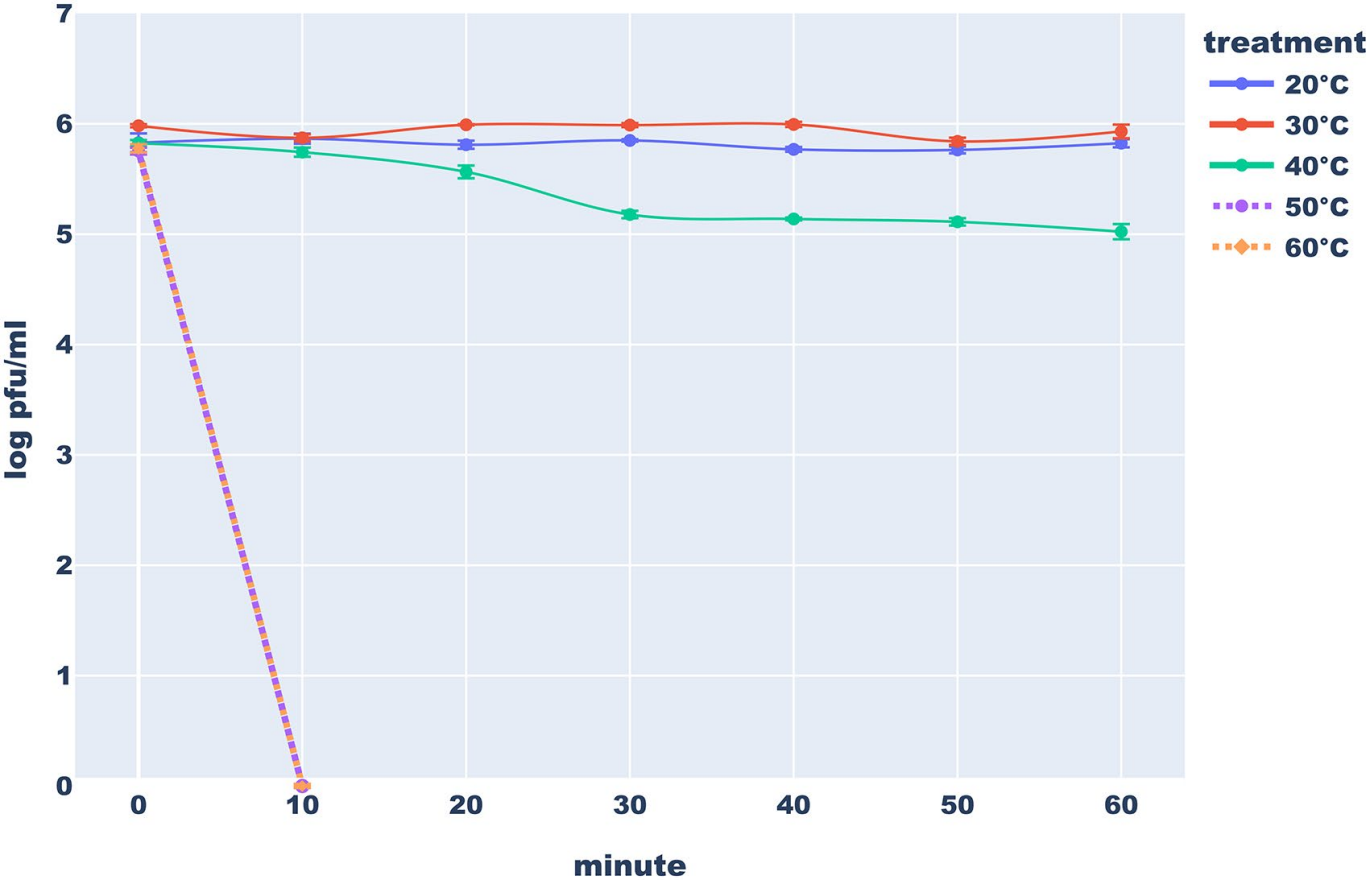
Heat stability of the Thurquoise phage. Results are expressed as log_10_ (PFU/mL). Data points represent the mean of three independent experiments and error bars show standard error of the mean (SEM) calculated from log-transformed data.

In the proper buffer, the studied phage seems to be stable in both refrigerator and freezer. On the other hand, Thurquoise is clearly sensitive to heating. It appears that the phage is stable in 20°C and 30°C but a significant drop of titer can be observed after just a half an hour in 40°C. Exposure of the phage to higher temperatures led to an immediate extinction. All differences in the titer between control (20°C) and other temperatures are statistically significant (Figure 11).

Similarly to the Thurquoise, phage SIOphi – a herellevirus infecting *B. subtilis*, is inactivated in 60°C though the process is markedly slower (Krasowska et al., 2015). Also a *B. cereus* BaceCM02 phage (that is unclassified but resembles Thurquoise morphologically) is sensitive to 50°C (Phongtang and Chukeatirote, 2021; Kazantseva et al., 2022). Only (Lee et al., 2014) claim that phages BPS10C, and BPS13 that are related to Thurquoise at the subfamily level, are stable at 50°C but fail to present raw data supporting this assertion.

Detailed results of all stability tests are presented in Supplementary Table 1.

In summary, we present the Thurquoise phage, a novel virus infecting *Bacillus thuringiensis* and the exemplar of the new candidate species in the *Caeruleovirus* genus in the *Bastillevirinae* subfamily of the *Herelleviridae* family. This phage has typical myophage morphology with an icosahedral head and contractile tail. Its genome and biology are also representative for herelleviruses (the complete annotated genome is available in genbank under accession number ON975036).

Additionally, our study demonstrates that proper buffer formulation and addition of cryoprotectants allow a safe long-term storage of viruses in common freezers and refrigerators.

## Data availability statement

The complete genome of the phage Thurquoise have been deposited in the NCBI GenBank database, accession number ON975036.

## Author contributions

MW and FW performed wet-lab experiments. JB performed the bioinformatic analysis and prepared figures. MW, JB, and GN contributed to the analysis of the data and wrote the manuscript. MŁ isolated and sequenced the genome of the phage, provided bacterial strains and supervised the project. All authors contributed to the article and approved the submitted version.

## Funding

This work was supported by: National Science Center (NCN, Poland) Sonata project 2016/23/D/NZ2/00435 (JB and MW). Adam Mickiewicz University Poznań, Poland, 29.020 BESTStudentGRANT II (FW). National Science Center (NCN, Poland) Preludium project 2015/17/N/NZ2/01830 (GN).

## Conflict of interest

GN was employed by genXone INC.

The remaining authors declare that the research was conducted in the absence of any commercial or financial relationships that could be construed as a potential conflict of interest.

## Publisher’s note

All claims expressed in this article are solely those of the authors and do not necessarily represent those of their affiliated organizations, or those of the publisher, the editors and the reviewers. Any product that may be evaluated in this article, or claim that may be made by its manufacturer, is not guaranteed or endorsed by the publisher.

## Abbreviations

TSB: Trypticase Soy Broth
TSA: Tryptone Soya Agar
EOP: Efficiency of plating assay
PFU: Plaque-forming unit
Cryo-EM: Cryogenic electron microscopy
MOI: Multiplicity of infection
SEM: Standard error of the mean
SM buffer: Suspension medium buffer

## References

[ref1] Ågren J. Sundström A. Håfström T. Segerman B. (2012). Gegenees: fragmented alignment of multiple genomes for determining phylogenomic distances and genetic signatures unique for specified target groups. PLoS One 7:e39107. doi: 10.1371/journal.pone.0039107, PMID: 22723939 PMC3377601

[ref2] Altschul S. F. Madden T. L. (1997). Gapped BLAST and PSI-BLAST: a new generation of protein database search programs. Nucleic Acids Res. 25, 3389–3402. doi: 10.1093/nar/25.17.3389, PMID: 9254694 PMC146917

[ref3] Armstrong A. M. Sobsey M. D. Casanova L. M. (2017). Disinfection of bacteriophage MS2 by copper in water. Appl. Microbiol. Biotechnol. 101, 6891–6897. doi: 10.1007/s00253-017-8419-x, PMID: 28756591

[ref4] Barylski J. Schuller M. B. P. Enault F. Dutilh B. E. Edwards R. A. Gillis A.. (2020). Analysis of Spounaviruses as a case study for the overdue reclassification of tailed phages. Syst. Biol. 69, 110–123. doi: 10.1093/sysbio/syz036, PMID: 31127947 PMC7409376

[ref5] Besemer J. Lomsadze A. Borodovsky M. (2001). GeneMarkS: a self-training method for prediction of gene starts in microbial genomes. Implications for finding sequence motifs in regulatory regions. Nucleic Acids Res. 29, 2607–2618. doi: 10.1093/nar/29.12.2607, PMID: 11410670 PMC55746

[ref6] Celandroni F. Mazzantini D. Salvetti S. Gueye S. A. Lupetti A. Senesi S.. (2016). Identification and pathogenic potential of clinical *Bacillus* and *Paenibacillus* isolates. PLoS One 11:e0152831. doi: 10.1371/journal.pone.0152831, PMID: 27031639 PMC4816569

[ref7] Chan P. P. Lowe T. M. (2019). tRNAscan-SE: searching for tRNA genes in genomic sequences. Methods Mol. Biol. 1962, 1–14. doi: 10.1007/978-1-4939-9173-0_1, PMID: 31020551 PMC6768409

[ref8] Chernomor O. von Haeseler A. Minh B. Q. (2016). Terrace aware data structure for Phylogenomic inference from Supermatrices. Syst. Biol. 65, 997–1008. doi: 10.1093/sysbio/syw037, PMID: 27121966 PMC5066062

[ref9] Delcher A. L. Salzberg S. L. Bratke K. A. Powers E. C. (2007). Identifying bacterial genes and endosymbiont DNA with glimmer. Bioinformatics 23, 673–679. doi: 10.1093/bioinformatics/btm009, PMID: 17237039 PMC2387122

[ref10] Elshaghabee F. M. F. Rokana N. Gulhane R. D. Sharma C. Panwar H. (2017). *Bacillus* as potential probiotics: status, concerns, and future perspectives. Front. Microbiol. 8:1490. doi: 10.3389/fmicb.2017.01490 28848511 PMC5554123

[ref11] Fokine A. Mesyanzhinov V. V. Chipman P. R. Leiman P. G. Rao V. B. Rossmann M. G. (2004). Molecular architecture of the prolate head of bacteriophage T4. Proc. Natl. Acad. Sci. U. S. A. 101, 6003–6008. doi: 10.1073/pnas.0400444101, PMID: 15071181 PMC395913

[ref12] Guerrero-Ferreira R. C. Hupfeld M. Nazarov S. Taylor N. M. I. Shneider M. M. Obbineni J. M.. (2019). Structure and transformation of bacteriophage A511 baseplate and tail upon infection of *Listeria* cells. EMBO J. 38:e99455. doi: 10.15252/embj.201899455 30606715 PMC6356063

[ref13] Guo F.-B. Zhang C.-T. (2006). ZCURVE_V: a new self-training system for recognizing protein-coding genes in viral and phage genomes. BMC Bioinformatics 7:9. doi: 10.1186/1471-2105-7-9, PMID: 16401352 PMC1352377

[ref14] Harper D. R. Abedon S. T. Burrowes B. H. McConville M. L.. (2021) Bacteriophages: Biology, Technology, Therapy. Cham. Springer International Publishing.

[ref15] Hock L. Gillis A. Leprince A. Tournay M. Mahillon J. (2019). Biocontrol potential of phage deep-blue against psychrotolerant *Bacillus weihenstephanensis*. Food Control 102, 94–103. doi: 10.1016/j.foodcont.2019.03.014

[ref16] Huang L. Xiang Y. (2020). Structures of the tailed bacteriophages that infect gram-positive bacteria. Curr. Opin. Virol. 45, 65–74. doi: 10.1016/j.coviro.2020.09.002, PMID: 33142120

[ref17] Hyatt D. Land M. L. Chen G. L. LoCascio P. F. Larimer F. W. Hauser L. J. (2010). Prodigal: prokaryotic gene recognition and translation initiation site identification. BMC Bioinformatics 11:119. doi: 10.1186/1471-2105-11-119, PMID: 20211023 PMC2848648

[ref18] Hyman P. (2019). Phages for phage therapy: isolation, characterization, and host range breadth. Pharmaceuticals 12:35. doi: 10.3390/ph12010035 30862020 PMC6469166

[ref19] Jones P. Fraser M. Binns D. Chang H. Y. Li W. McAnulla C.. (2014). InterProScan 5: genome-scale protein function classification. Bioinformatics 30, 1236–1240. doi: 10.1093/bioinformatics/btu031, PMID: 24451626 PMC3998142

[ref20] Kazantseva O. A. Piligrimova E. G. Shadrin A. M. (2022). Novel *Bacillus*-infecting bacteriophage B13–the founding member of the proposed new genus Bunatrivirus. Viruses 14:2300. doi: 10.3390/v14102300, PMID: 36298855 PMC9610010

[ref21] Klumpp J. Ackermann H. W. Lavigne R. Loessner M. J. (2010). The SPO1-related bacteriophages. Arch. Virol. 155, 1547–1561. doi: 10.1007/s00705-010-0783-0, PMID: 20714761

[ref22] Krasowska A. Biegalska A. Augustyniak D. Łoś M. Richert M. Łukaszewicz M. (2015). Isolation and characterization of phages infecting *Bacillus subtilis*. Biomed. Res. Int. 2015:179597. doi: 10.1155/2015/179597 26273592 PMC4529890

[ref23] Lee J.-H. Shin H. Ryu S. (2014). Characterization and comparative genomic analysis of bacteriophages infecting members of the *Bacillus cereus* group. Arch. Virol. 159, 871–884. doi: 10.1007/s00705-013-1920-3, PMID: 24264384

[ref24] Li J. Dennehy J. J. (2011). Differential bacteriophage mortality on exposure to copper. Appl. Environ. Microbiol. 77, 6878–6883. doi: 10.1128/AEM.05661-11, PMID: 21841029 PMC3187089

[ref25] Marchler-Bauer A. Bryant S. H. (2004). CD-search: protein domain annotations on the fly. Nucleic Acids Res. 32, W327–W331. doi: 10.1093/nar/gkh454, PMID: 15215404 PMC441592

[ref26] Nawrocki E. P. Eddy S. R. (2013). Infernal 1.1: 100-fold faster RNA homology searches. Bioinformatics 29, 2933–2935. doi: 10.1093/bioinformatics/btt509, PMID: 24008419 PMC3810854

[ref27] Nguyen L.-T. Minh B. Q. Schmidt H. A. von Haeseler A. (2015). IQ-TREE: a fast and effective stochastic algorithm for estimating maximum-likelihood phylogenies. Mol. Biol. Evol. 32, 268–274. doi: 10.1093/molbev/msu300, PMID: 25371430 PMC4271533

[ref28] Nishimura Y. Uehara H. Yoshida T. Kuronishi M. Ogata H. Goto S. (2017). ViPTree: the viral proteomic tree server. Bioinformatics 33, 2379–2380. doi: 10.1093/bioinformatics/btx157, PMID: 28379287

[ref29] Olson M. R. Axler R. P. Hicks R. E. (2004). Effects of freezing and storage temperature on MS2 viability. J. Virol. Methods 122, 147–152. doi: 10.1016/j.jviromet.2004.08.010, PMID: 15542138

[ref30] Ozaki T. Suzuki A. Abe N. Kimura K. Kaneko J. (2017). Genomic analysis of *Bacillus subtilis* lytic bacteriophage ϕNIT1 capable of obstructing natto fermentation carrying genes for the capsule-lytic soluble enzymes poly-γ-glutamate hydrolase and levanase. Biosci. Biotechnol. Biochem. 81, 135–146. doi: 10.1080/09168451.2016.1232153, PMID: 27885938

[ref31] Phongtang W. Chukeatirote E. (2021). Incidence and characterisation of *Bacillus cereus* bacteriophages from Thua Nao, a Thai fermented soybean product. Biomol. Concepts 12, 85–93. doi: 10.1515/bmc-2021-0009, PMID: 34218551

[ref32] Raza S. Folga M. Łoś M. Foltynowicz Z. Paczesny J. (2022). The effect of zero-Valent iron nanoparticles (nZVI) on bacteriophages. Viruses 14:867. doi: 10.3390/v14050867, PMID: 35632609 PMC9144403

[ref33] Remize F. (2017) ‘Spore-Forming Bacteria, the Microbiological Quality of Food. Amsterdam, Netherlands: Elsevier. pp. 99–120.

[ref34] Rohwer F. Edwards R. (2002). The phage proteomic tree: a genome-based taxonomy for phage. J. Bacteriol. 184, 4529–4535. doi: 10.1128/JB.184.16.4529-4535.2002, PMID: 12142423 PMC135240

[ref35] Samuelson J. C. Zhu Z. Xu S.-Y. (2004). The isolation of strand-specific nicking endonucleases from a randomized SapI expression library. Nucleic Acids Res. 32, 3661–3671. doi: 10.1093/nar/gkh674, PMID: 15247348 PMC484165

[ref36] Shin H. Ryu S. Bandara N. Shin E. Kim K. P. (2011). Prevalence of *Bacillus cereus* bacteriophages in fermented foods and characterization of phage JBP901. Res. Microbiol. 162, 791–797. doi: 10.1016/j.resmic.2011.07.001, PMID: 21810470

[ref37] Thorne C. B. Holt S. C. (1974). Cold lability of *Bacillus cereus* bacteriophage CP-51. J. Virol. 14, 1008–1012. doi: 10.1128/jvi.14.4.1008-1012.1974, PMID: 4138063 PMC355608

[ref38] Vallat R. (2018). Pingouin: statistics in python. J Open Source Softw. 3:1026. doi: 10.21105/joss.01026

[ref39] Willms I. M. Hoppert M. Hertel R. (2017). Characterization of *Bacillus subtilis* viruses vB_BsuM-Goe2 and vB_BsuM-Goe3. Viruses 9:146. doi: 10.3390/v9060146, 9, PMID: 28604650 PMC5490822

